# Supplementary figures and images for: Molecular diet analysis enables detection of diatom and cyanobacteria DNA in the gut of Macoma balthica
Source: PLoS One. 2022 Nov 23;17(11):e0278070. doi: 10.1371/journal.pone.0278070 (PMC9683582; doi:10.1371/journal.pone.0278070)

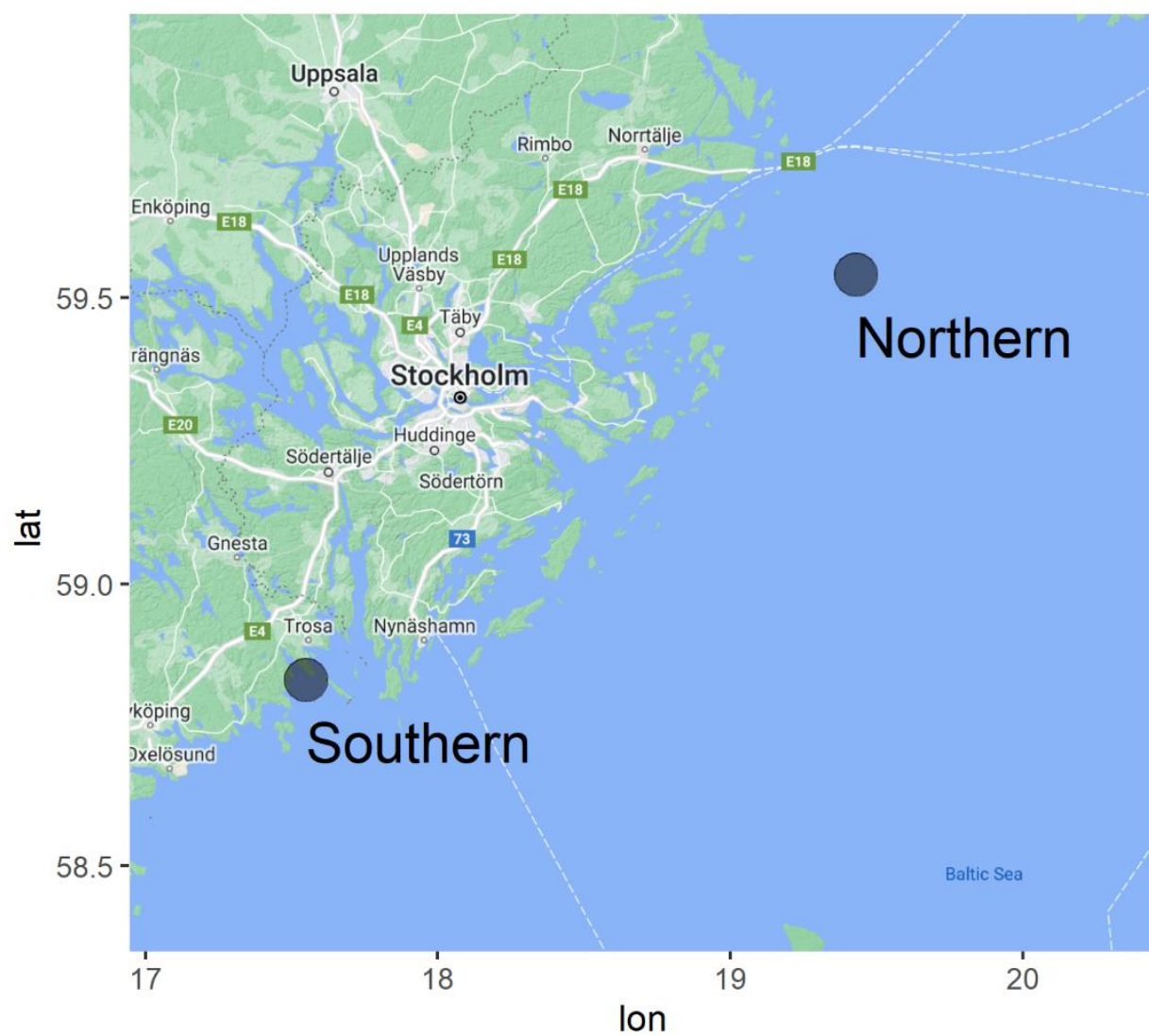

Supplement: S1 Fig — Map of northern and southern Stockholm archipelago stations where Macoma balthica were collected to be utilized in the feeding experiment. (PDF) [file pone.0278070.s001.pdf]

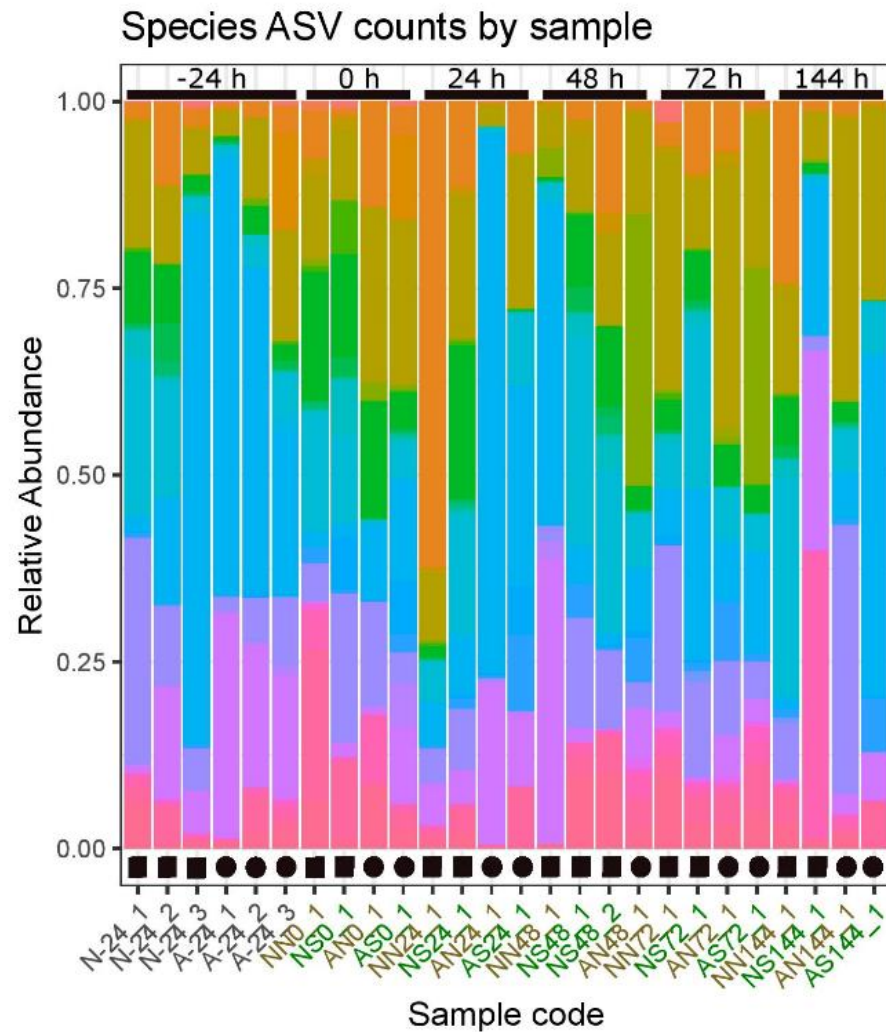

## Species

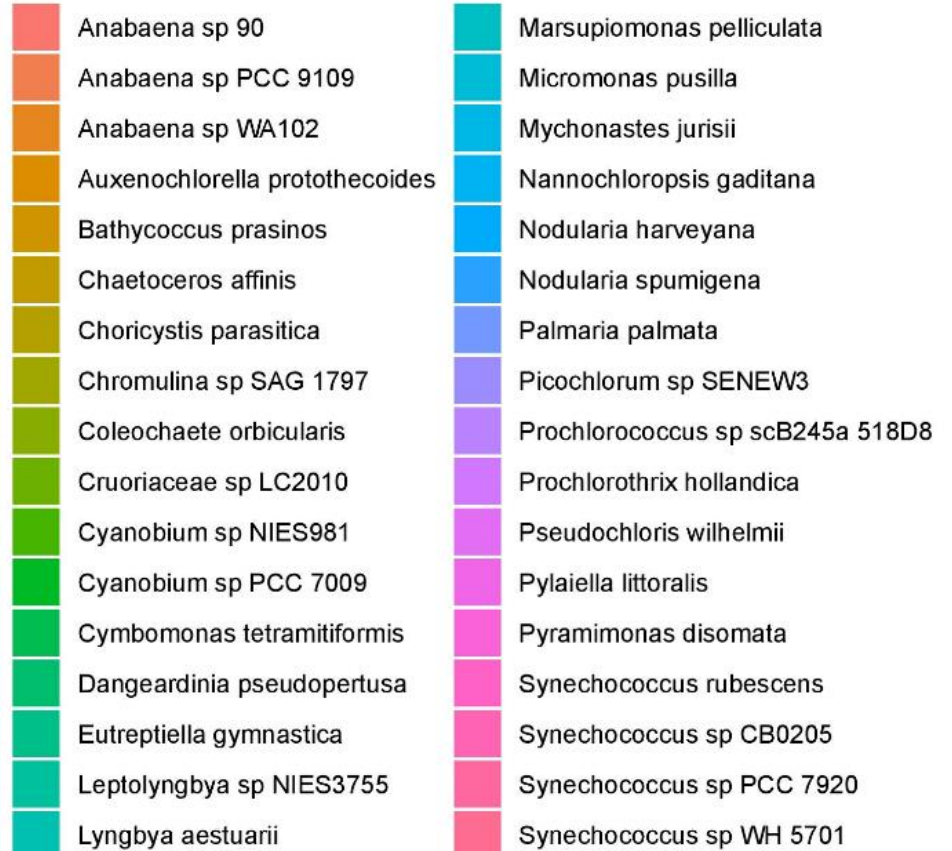

Supplement: S2 Fig — Relative abundance of all species found with DNA metabarcoding of the 23S rRNA gene in Macoma balthica guts. Sample name colors indicate the feeding treatment, where gray indicates no feeding, green the diatom Skeletonema marinoi and brown the cyanobacteria Nodularia spumigena. The shape above the sample name indicates the region of M. balthica, where square represents the northern Stockholm archipelago clams and circle the southern Stockholm archipelago clams. The black bars above the relative abundance indicates the time point when the samples were taken in hours, with -24 h being before the feeding began, 0 h directly after feeding, and 24 h, 48 h, 72 h, and 144 h indicating 1, 2, 3, and 7 days after feeding, respectively. (PDF) [file pone.0278070.s002.pdf]

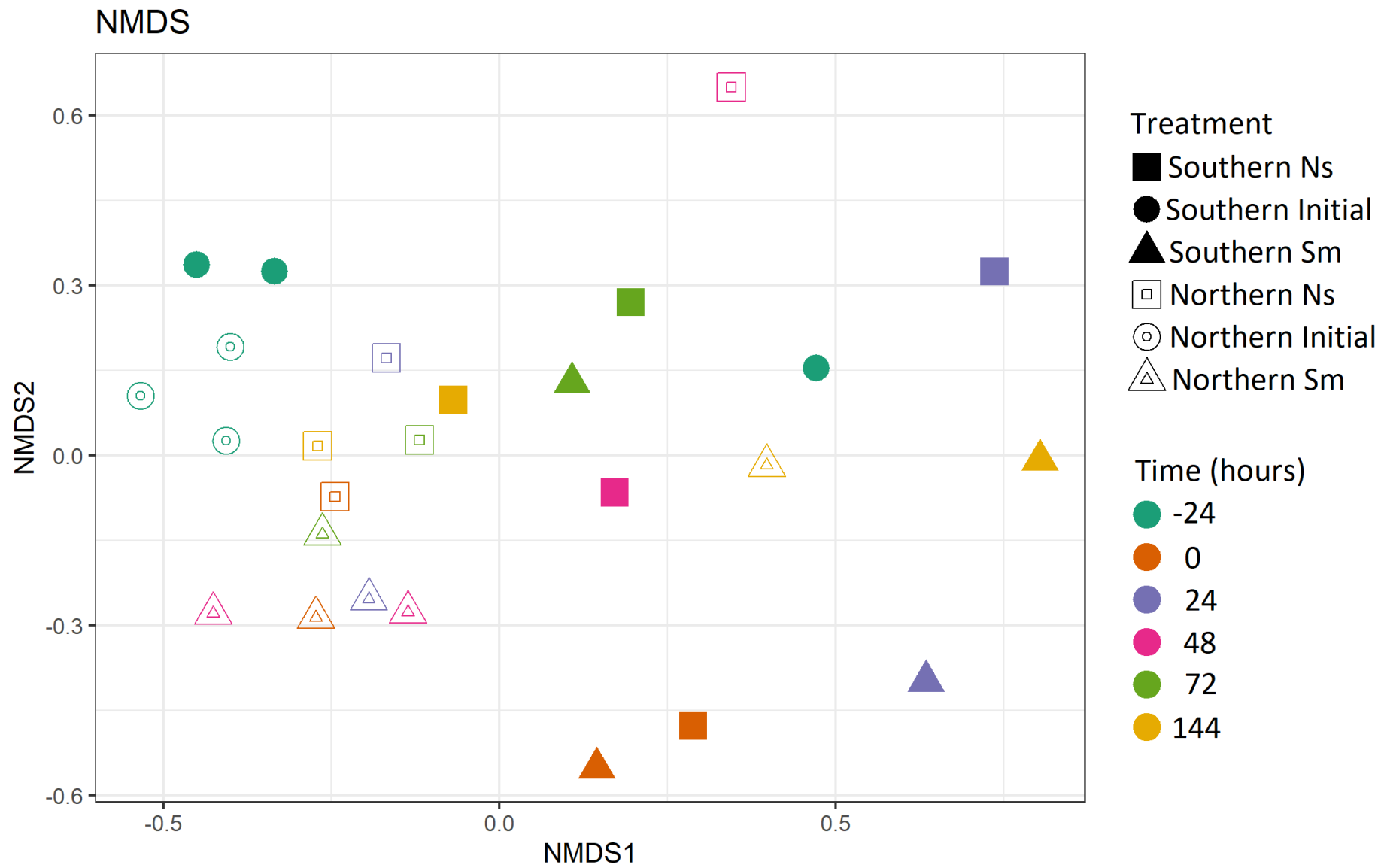

Supplement: S3 Fig — NMDS using Bray-Curtis distance on DNA metabarcoding data from the guts of Macoma balthica, with filled shapes representing the southern Stockholm archipelago clams, and open shapes representing the northern Stockholm archipelago clams. Samples taken before the experiment began (Initial, circles) weren’t fed, while squares represent feeding of the cyanobacteria Nodularia spumigena (Ns), and triangles the diatom Skeletonema marinoi (Sm) (stress = 0.16). Colors are the sampling time point in hours. (PDF) [file pone.0278070.s003.pdf]
