# Supplementary material for: Molecular diet analysis enables detection of diatom and cyanobacteria DNA in the gut of Macoma balthica
Source: PLoS One. 2022 Nov 23;17(11):e0278070. doi: 10.1371/journal.pone.0278070 (PMC9683582; doi:10.1371/journal.pone.0278070)
Supplement: S4 Fig — Phytoplankton consumption by M. balthica region (northern A, B; southern C, D), with (dashed lines) and without (solid or dotted lines) gut clearing, determined by qPCR and reported in number of copies μL-1. Panels A and C represents Skeletonema marinoi (green), while B and D represents consumption of Nodularia spumigena (brown). Squares and solid lines represent the northern Stockholm archipelago clams, and circles and dotted lines represent the southern Stockholm archipelago clams. Error bars represent standard error. (PDF) [file pone.0278070.s004.pdf]

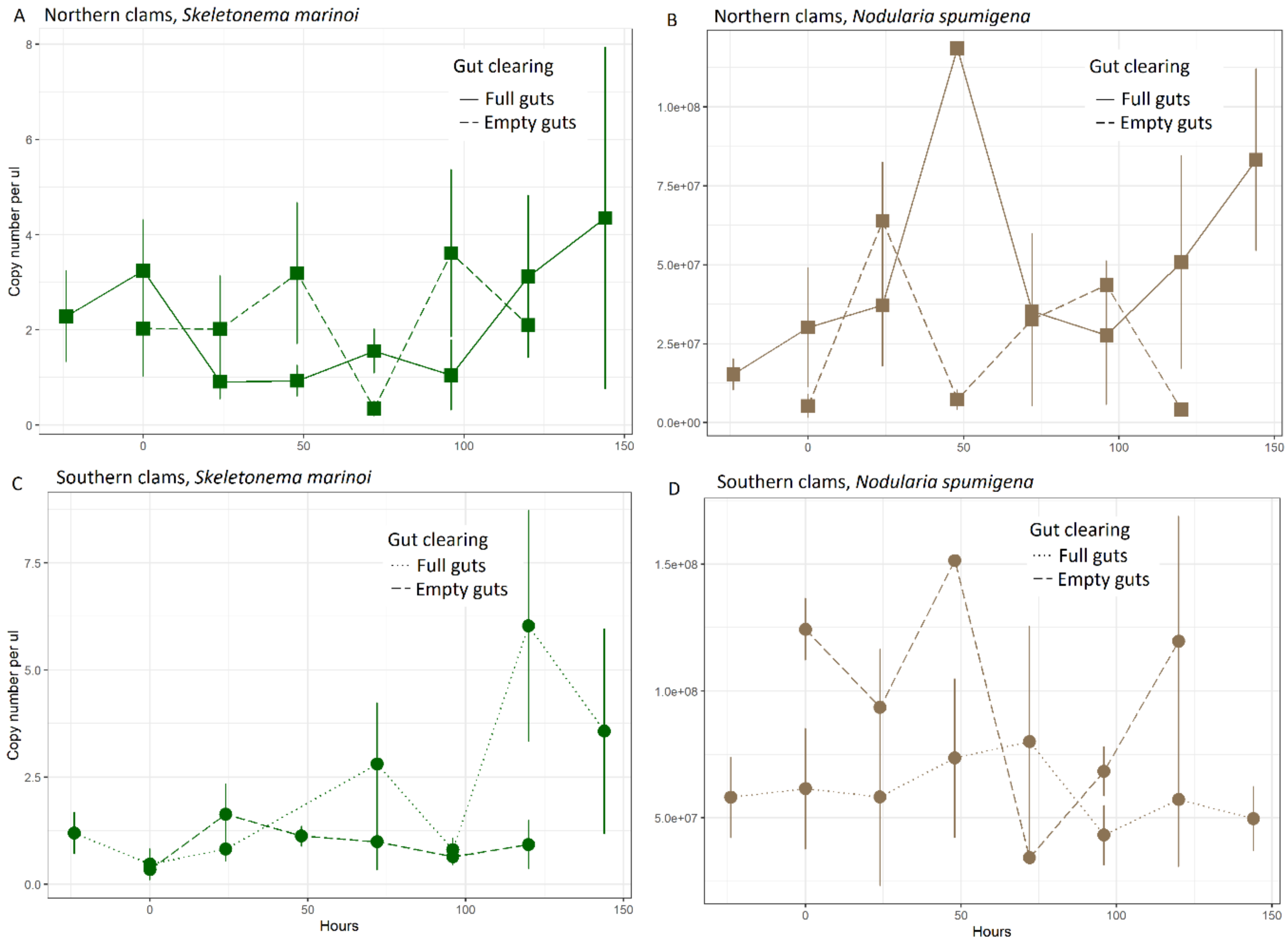
A
Northern clams, *Skeletonema marinoi*
Copy number per ul
Gut clearing
Full guts
Empty guts
B
Northern clams, *Nodularia spumigena*
Gut clearing
Full guts
Empty guts
C
Southern clams, *Skeletonema marinoi*
Copy number per ul
Gut clearing
Full guts
Empty guts
Hours
D
Southern clams, *Nodularia spumigena*
Gut clearing
Full guts
Empty guts
Hours
