# Supplementary material for: Molecular diet analysis enables detection of diatom and cyanobacteria DNA in the gut of Macoma balthica
Source: PLoS One. 2022 Nov 23;17(11):e0278070. doi: 10.1371/journal.pone.0278070 (PMC9683582; doi:10.1371/journal.pone.0278070)
Supplement: S1 Table — Sample is the Macoma balthica individual, region refers to the origin of the individual, phytoplankton fed refers to the phytoplankton culture fed to the individual, empty guts refers to the treatment where the individuals were placed in fresh water for 24 hours to empty their guts, time hours refers to the number of hours after feeding that the individuals were sampled, biological replicate refers to the individual replicate number, wet weight g refers to the wet weight of the individual in grams, CT AVG refers to the average Ct value of the three technical replicates from the individual, CT SD refers to the standard deviation of the three technical replicates Ct value from the individual, Log DNA refers to the log(10) amount of DNA detected from the qPCR assays, No copies refers to the number of copies of the target amplicon DNA, Copy no per μl refers to the number of copies of the target amplicon DNA standardized to the total DNA concentration, and sequenced refers to the selected samples which were also analyzed for next-generation sequencing to detect all phytoplankton in the guts, for more information on these samples see S2 Table. (PDF) [file pone.0278070.s005.pdf]

Sample	Region	Phytoplankton fed	Empty guts	Time hours	Biological replicate	Wet Weight g	CT AVG	CT SD	Log DNA	No copies	Copy no Per µl	Sequenced?
N_-1_1	Northern	None	No	-24	1	0,1	28,5485655	0,08357906	6,3821347	1,12E+08	24665067,92	Yes
N_-1_2	Northern	None	No	-24	2	0,1	28,7049828	0,66302764	6,3346145	1,04E+08	13265148,69	Yes
N_-1_3	Northern	None	No	-24	3	0,1	29,6121375	0,6418429	6,0590177	66684083	8002089,932	Yes
A_-1_1	Southern	None	No	-24	1	0,1	27,3457667	0,13250558	6,7475493	1,96E+08	41084685,07	Yes
A_-1_2	Southern	None	No	-24	2	0,1	25,3022156	0,49619982	7,3683875	4,72E+08	89635118,16	Yes
A_-1_3	Southern	None	No	-24	3	0,1	27,7764231	0,27951479	6,6167143	1,61E+08	43429777,8	Yes
NN_0_1	Northern	*Nodularia spumigena*	No	0	1	0,2	29,1578935	0,39073899	6,1970186	83527217	1020888,211	Yes
NN_0_2	Northern	*Nodularia spumigena*	No	0	2	0,1	28,3728765	0,45915782	6,4355096	1,22E+08	24370219,69	No
NN_0_3	Northern	*Nodularia spumigena*	No	0	3	0,1	26,2448483	0,32437268	7,0820123	3,17E+08	65060985,84	No
AN_0_1	Southern	*Nodularia spumigena*	No	0	1	0,1	28,1555614	0,15904135	6,5015307	1,35E+08	16193377,59	Yes
AN_0_2	Southern	*Nodularia spumigena*	No	0	2	0,1	25,4253483	0,40562001	7,3309794	4,48E+08	96395872,34	No
AN_0_3	Southern	*Nodularia spumigena*	No	0	3	0,1	25,0079886	0,28861403	7,4577748	5,32E+08	71849937,19	No
NN_24_1	Northern	*Nodularia spumigena*	No	24	1	0,1	25,4829629	0,26258868	7,3134758	4,38E+08	72230771,64	Yes

NN_24_2	Northern	*Nodularia spumigena*	No	24	2	0,1	26,9402288	0,42731413	6,8707532	2,34E+08	33994721,51	No
NN_24_3	Northern	*Nodularia spumigena*	No	24	3	0,1	30,7809601	3,07859135	5,7039251	36453409	5285744,37	No
AN_24_1	Southern	*Nodularia spumigena*	No	24	1	0,1	26,5974083	0,25699148	6,9749033	2,73E+08	38151353,1	Yes
AN_24_2	Southern	*Nodularia spumigena*	No	24	2	0,1	29,4024639	5,0680995	6,1227172	74035845	10365018,29	No
AN_24_3	Southern	*Nodularia spumigena*	No	24	3	0,1	24,7647991	0,1205915	7,5316566	5,87E+08	126282615,4	No
NN_0E_1	Northern	*Nodularia spumigena*	Yes	0	1	0,1	28,5226771	0,77263516	6,3899997	1,14E+08	12485359,13	Yes
NN_0E_2	Northern	*Nodularia spumigena*	Yes	0	2	0,2	29,60864	2,31979132	6,0600802	66801115	1736828,994	No
NN_0E_3	Northern	*Nodularia spumigena*	Yes	0	3	0,1	29,7649104	0,52839494	6,0126047	61748515	1605461,39	No
AN_0E_1	Southern	*Nodularia spumigena*	Yes	0	1	0,1	24,1724701	0,85767031	7,7116083	7,44E+08	100411485,9	Yes
AN_0E_2	Southern	*Nodularia spumigena*	Yes	0	2	0,1	25,1691717	0,51005244	7,4088068	4,98E+08	132047003,9	No

AN_0E_3	Southern	*Nodularia spumigena*	Yes	0	3	0,1	25,1531258	0,21387686	7,4136816	5,02E+08	140442099,2	No
NN_48_1	Northern	*Nodularia spumigena*	No	48	1	0,1	24,8032621	0,09490024	7,5199714	5,78E+08	118553877,1	Yes
AN_48_1	Southern	*Nodularia spumigena*	No	48	1	0,1	24,6491337	0,80548078	7,5667962	6,15E+08	58458001,83	Yes
AN_48_2	Southern	*Nodularia spumigena*	No	48	2	0,1	30,671978	0,24445805	6,552815	1,46E+08	28465164,29	No
AN_48_3	Southern	*Nodularia spumigena*	No	48	3	0,1	28,8157209	0,15023141	7,1194381	3,35E+08	133818808,4	No
NN_24E_1	Northern	*Nodularia spumigena*	Yes	24	1	0,1	29,3734328	0,21159674	6,9491963	2,63E+08	94547123,9	No
NN_24E_2	Northern	*Nodularia spumigena*	Yes	24	2	0,1	28,2945506	0,10931833	7,2785255	4,17E+08	66765797,41	No
NN_24E_3	Northern	*Nodularia spumigena*	Yes	24	3	0,1	28,367979	0,24090935	7,2561114	4,05E+08	30345945,99	No
AN_24E_2	Southern	*Nodularia spumigena*	Yes	24	2	0,1	29,1604939	0,23862052	7,014196	2,88E+08	70622822,08	No
AN_24E_3	Southern	*Nodularia spumigena*	Yes	24	3	0,1	28,7748763	0,17631967	7,1319059	3,4E+08	116434625	No

NN_72_1	Northern	*Nodularia spumigena*	No	72	1	0,1	30,4152902	0,1608346	6,631169	1,64E+08	32057939,63	Yes
NN_72_2	Northern	*Nodularia spumigena*	No	72	2	0,1	31,8277651	0,50560713	6,2000106	83931377	30215295,65	No
NN_72_3	Northern	*Nodularia spumigena*	No	72	3	0,1	30,7582906	1,59546077	6,5264681	1,4E+08	43465418,83	No
AN_72_1	Southern	*Nodularia spumigena*	No	72	1	0,1	27,9000301	0,25738087	7,3989529	4,92E+08	169637456,3	Yes
AN_72_2	Southern	*Nodularia spumigena*	No	72	2	0,1	30,4279989	1,35103273	6,6272897	1,63E+08	22881664,11	No
AN_72_3	Southern	*Nodularia spumigena*	No	72	3	0,2	28,3231297	0,28109118	7,2698017	4,12E+08	47415843,73	No
NN_48E_1	Northern	*Nodularia spumigena*	Yes	48	1	0,3	34,0659688	0,83851707	5,5167983	26113858	1479785,309	No
NN_48E_2	Northern	*Nodularia spumigena*	Yes	48	2	0,2	30,0889111	0,25550053	6,7307964	1,91E+08	10973176,63	No
NN_48E_3	Northern	*Nodularia spumigena*	Yes	48	3	0,2	30,1147168	0,34200549	6,7229192	1,89E+08	9430807,76	No
AN_48E_1	Southern	*Nodularia spumigena*	Yes	48	1	0,1	28,1406345	0,04895086	7,3255084	4,45E+08	151306161,6	No

NN_96_1	Northern	*Nodularia spumigena*	No	96	1	0,1	29,3247948	0,26417425	6,9640431	2,68E+08	71098491	No
NN_96_2	Northern	*Nodularia spumigena*	No	96	2	0,1	31,4864788	0,10164191	6,3041884	99149798	10410728,8	No
NN_96_3	Northern	*Nodularia spumigena*	No	96	3	0,1	32,8610967	0,08011846	5,8845859	49791940	1593342,066	No
AN_96_1	Southern	*Nodularia spumigena*	No	96	1	0,1	28,8955186	0,22554536	7,0950798	3,23E+08	30711175,59	No
AN_96_2	Southern	*Nodularia spumigena*	No	96	2	0,1	29,7103532	0,28016728	6,8463513	2,26E+08	66744241,28	No
AN_96_3	Southern	*Nodularia spumigena*	No	96	3	0,1	29,6797295	0,28481835	6,8556992	2,29E+08	32110389,27	No
NN_72E_1	Northern	*Nodularia spumigena*	Yes	72	1	0,2	30,2691797	0,26131144	6,6757693	1,76E+08	10108368,52	No
NN_72E_2	Northern	*Nodularia spumigena*	Yes	72	2	0,3	30,9590594	0,17943786	6,4651833	1,28E+08	1043898,496	No
NN_72E_3	Northern	*Nodularia spumigena*	Yes	72	3	0,2	29,8865859	0,77701968	6,7925562	2,09E+08	86772179,04	No
AN_72E_1	Southern	*Nodularia spumigena*	Yes	72	1	0,1	29,235068	0,1151282	6,742431	1,94E+08	34949231,24	No

AN_72E_2	Southern	*Nodularia spumigena*	Yes	72	2	0,1	29,206501	0,19489539	6,7508878	1,97E+08	37356183,7	No
AN_72E_3	Southern	*Nodularia spumigena*	Yes	72	3	0,1	29,0807826	0,34652898	6,7881046	2,08E+08	30639191,14	No
NN_120_1	Northern	*Nodularia spumigena*	No	120	1	0,1	30,0404421	0,4125419	6,5040136	1,35E+08	27092206,99	No
NN_120_2	Northern	*Nodularia spumigena*	No	120	2	0,1	28,950922	0,14821513	6,8265477	2,2E+08	8352084,152	No
NN_120_3	Northern	*Nodularia spumigena*	No	120	3	0,1	26,7612311	0,05167426	7,4747688	5,44E+08	117062038,5	No
AN_120_1	Southern	*Nodularia spumigena*	No	120	1	0,1	26,9803314	0,28811032	7,4099078	4,99E+08	104796636	No
AN_120_2	Southern	*Nodularia spumigena*	No	120	2	0,1	29,6111666	0,29910338	6,6310934	1,64E+08	13150474,1	No
AN_120_3	Southern	*Nodularia spumigena*	No	120	3	0,1	29,1804695	0,09408595	6,758594	1,99E+08	53694195,53	No
NN_96E_1	Northern	*Nodularia spumigena*	Yes	96	1	0,1	28,5016543	0,16895905	6,9595458	2,67E+08	58645088,97	No
NN_96E_2	Northern	*Nodularia spumigena*	Yes	96	2	0,1	29,036267	0,14768277	6,8012827	2,12E+08	38122448,76	No

NN_96E_3	Northern	*Nodularia spumigena*	Yes	96	3	0,1	28,2530905	0,1208026	7,0331289	2,96E+08	34055214,27	No
AN_96E_1	Southern	*Nodularia spumigena*	Yes	96	1	0,1	27,8985049	0,44102404	7,138098	3,43E+08	68683919,19	No
AN_96E_2	Southern	*Nodularia spumigena*	Yes	96	2	0,1	28,2766412	0,07917611	7,0261571	2,93E+08	85030861,31	No
AN_96E_3	Southern	*Nodularia spumigena*	Yes	96	3	0,1	29,2847608	0,43986604	6,7277203	1,9E+08	51291222,16	No
NN_144_1	Northern	*Nodularia spumigena*	No	144	1	0,1	28,7902877	0,09881457	6,8741008	2,36E+08	25915024,17	Yes
NN_144_2	Northern	*Nodularia spumigena*	No	144	2	0,1	27,0447013	0,17767307	7,3908522	4,86E+08	106996077,7	No
NN_144_3	Northern	*Nodularia spumigena*	No	144	3	0,1	28,6658065	0,25951907	6,9109513	2,49E+08	116808970	No
AN_144_1	Southern	*Nodularia spumigena*	No	144	1	0,1	28,1527996	0,2519933	7,0628184	3,09E+08	37064796,01	Yes
AN_144_2	Southern	*Nodularia spumigena*	No	144	2	0,1	28,3613733	0,08513179	7,0010736	2,83E+08	62239935,57	No
NN_120E_1	Northern	*Nodularia spumigena*	Yes	120	1	0,3	30,0530459	0,14004678	6,5002824	1,35E+08	2501310,392	No

NN_120E_2	Northern	*Nodularia spumigena*	Yes	120	2	0,2	30,3142033	0,08687773	6,4229712	1,19E+08	2389955,129	No
NN_120E_3	Northern	*Nodularia spumigena*	Yes	120	3	0,1	31,9913387	3,74199462	5,9264835	53452842	7483397,825	No
AN_120E_1	Southern	*Nodularia spumigena*	Yes	120	1	0,1	29,3786812	3,02097869	6,6999168	1,82E+08	38274838,38	No
AN_120E_2	Southern	*Nodularia spumigena*	Yes	120	2	0,1	25,4462833	0,12222711	7,8640369	9,05E+08	208057446,6	No
AN_120E_3	Southern	*Nodularia spumigena*	Yes	120	3	0,1	27,6017431	0,48552674	7,2259494	3,88E+08	112550423,8	No
N_-1_1	Northern	None	No	-24	1	0,1	28,8049456	0,64734894	1,0218686	10,51644	2,313616224	Yes
N_-1_2	Northern	None	No	-24	2	0,1	27,2288901	0,07809111	1,4866584	30,66609	3,909926237	Yes
N_-1_3	Northern	None	No	-24	3	0,1	29,8362115	0,40483612	0,7177412	5,220849	0,62650191	Yes
A_-1_1	Southern	None	No	-24	1	0,1	29,5813046	0,35228962	0,792915	6,207475	1,303569792	Yes
A_-1_2	Southern	None	No	-24	2	0,1	31,5771246	7,8631115	0,2043338	1,600788	0,30414969	Yes
A_-1_3	Southern	None	No	-24	3	0,1	29,3468316	0,51708829	0,8620627	7,278849	1,965289119	Yes
NS_0_1	Northern	*Skeletonema marinoi*	No	0	1	0,1	28,364831	0,2536431	1,1516615	14,17952	5,104627023	Yes
NS_0_2	Northern	*Skeletonema marinoi*	No	0	2	0,1	29,1553891	0,24863546	0,9185204	8,289349	3,191399238	No
NS_0_3	Northern	*Skeletonema marinoi*	No	0	3	0,1	30,3794797	1,03561938	0,5575276	3,610169	1,44406776	No

AS_0_1	Southern	*Skeletonema marinoi*	No	0	1	0,1	29,2176711	0,57972753	0,900153	7,946082	1,191912277	Yes
AS_0_2	Southern	*Skeletonema marinoi*	No	0	2	0,1	32,9424756	0,54332906	-0,198318	0,633406	0,107679041	No
AS_0_3	Southern	*Skeletonema marinoi*	No	0	3	0,1	32,3594424	1,10553014	-0,026377	0,941072	0,094107192	No
NS_24_1	Northern	*Skeletonema marinoi*	No	24	1	0,1	28,9030539	0,24115165	0,9929358	9,838657	0,787092564	Yes
NS_24_2	Northern	*Skeletonema marinoi*	No	24	2	0,1	32,1297919	1,46525395	0,0413483	1,099888	0,357463503	No
NS_24_3	Northern	*Skeletonema marinoi*	No	24	3	0,2	28,3384209	0,10002762	1,15945	14,43611	1,587971569	No
AS_24_1	Southern	*Skeletonema marinoi*	No	24	1	0,1	29,2749958	0,36859009	0,8832476	7,642713	1,069979874	Yes
AS_24_2	Southern	*Skeletonema marinoi*	No	24	2	0,1	31,3912398	1,29173481	0,2591525	1,816153	0,27242299	No
AS_24_3	Southern	*Skeletonema marinoi*	No	24	3	0,1	30,0110124	0,68302226	0,6661912	4,63651	1,112762296	No
NS_0E_1	Northern	*Skeletonema marinoi*	Yes	0	1	0,1	28,646904	0,43619326	1,0684762	11,70782	2,926956222	Yes

NS_0E_2	Northern	*Skeletonema marinoi*	Yes	0	2	0,1	27,7365017	0,140378	1,3369602	21,72502	3,113919565	No
NS_0E_3	Northern	*Skeletonema marinoi*	Yes	0	3	0,3	31,0167592	1,9597379	0,3695894	2,342014	0,046840273	No
AS_0E_1	Southern	*Skeletonema marinoi*	Yes	0	1	0,1	31,8623352	0	0,1202232	1,318934	0,184650811	Yes
AS_0E_2	Southern	*Skeletonema marinoi*	Yes	0	2	0,1	31,039362	0,99046177	0,3629237	2,306342	0,403609862	No
AS_0E_3	Southern	*Skeletonema marinoi*	Yes	0	3	0,1	31,4111754	0,53310561	0,2532733	1,791733	0,430015973	No
NS_48_1	Northern	*Skeletonema marinoi*	No	48	1	0,2	27,2282022	0,40285194	1,4868613	30,68042	1,43175277	Yes
NS_48_2	Northern	*Skeletonema marinoi*	No	48	2	0,2	29,2682845	0,92689198	0,8852268	7,677623	1,036479121	Yes
NS_48_3	Northern	*Skeletonema marinoi*	No	48	3	0,2	33,6031176	0,33379292	0,8708467	7,427568	0,321861292	No
NS_24E_2	Northern	*Skeletonema marinoi*	Yes	24	2	0,1	32,0402781	1,30372691	1,3347349	21,61399	3,134028227	No
NS_24E_3	Northern	*Skeletonema marinoi*	Yes	24	3	0,2	31,1063054	0,16456623	1,6119604	40,92233	0,90029134	No

AS_24E_1	Southern	*Skeletonema marinoi*	Yes	24	1	0,2	32,5884336	0,2282283	1,1720292	14,86036	0,928772247	No
AS_24E_2	Southern	*Skeletonema marinoi*	Yes	24	2	0,1	33,2417374	0,35038209	0,978113	9,508521	2,329587706	No
NS_72_1	Northern	*Skeletonema marinoi*	No	72	1	0,1	30,7595469	0,17298701	1,7148866	51,86646	2,074658547	Yes
NS_72_2	Northern	*Skeletonema marinoi*	No	72	2	0,1	31,8913066	0,35621592	1,3789532	23,93058	0,622195091	No
NS_72_3	Northern	*Skeletonema marinoi*	No	72	3	0,1	31,4898281	0,3209371	1,4981217	31,4863	1,967893914	No
AS_72_1	Southern	*Skeletonema marinoi*	No	72	1	0,1	31,6129773	0,20702209	1,461568	28,94463	5,64420306	Yes
AS_72_2	Southern	*Skeletonema marinoi*	No	72	2	0,1	34,1306203	0,39862943	0,7142712	5,179302	1,243032385	No
AS_72_3	Southern	*Skeletonema marinoi*	No	72	3	0,1	33,1906268	0,32076767	0,9932838	9,846544	1,526214331	No
NS_48E_1	Northern	*Skeletonema marinoi*	Yes	48	1	0,1	32,2348347	0,42198282	1,2769859	18,92282	6,055302287	No
NS_48E_2	Northern	*Skeletonema marinoi*	Yes	48	2	0,1	34,0672112	0,19956732	0,7330926	5,408696	1,108782659	No

NS_48E_3	Northern	*Skeletonema marinoi*	Yes	48	3	0,1	32,9383901	0,09151177	1,0681537	11,69913	2,398322531	No
AS_48E_1	Southern	*Skeletonema marinoi*	Yes	48	1	0,1	34,6773733	0,43149269	0,5519818	3,564362	1,21188312	No
AS_48E_2	Southern	*Skeletonema marinoi*	Yes	48	2	0,1	33,4202601	0,30119514	0,9251231	8,416338	1,472859084	No
AS_48E_3	Southern	*Skeletonema marinoi*	Yes	48	3	0,1	34,3339704	0,09005885	0,653912	4,507254	0,676088088	No
NS_96_1	Northern	*Skeletonema marinoi*	No	96	1	0,2	32,8870786	0,42326832	1,0833842	12,1167	0,315034093	No
NS_96_2	Northern	*Skeletonema marinoi*	No	96	2	0,3	31,2369925	0,29471928	1,5731693	37,42565	0,30620985	No
NS_96_3	Northern	*Skeletonema marinoi*	No	96	3	0,1	33,8921839	0,35405156	0,7850448	6,095998	2,529839301	No
AS_96_1	Southern	*Skeletonema marinoi*	No	96	1	0,1	34,7298088	0,59057963	0,5364177	3,438885	0,962887879	No
AS_96_2	Southern	*Skeletonema marinoi*	No	96	2	0,1	33,8809141	0,47871867	0,78839	6,143134	1,16719552	No
AS_96_3	Southern	*Skeletonema marinoi*	No	96	3	0,2	34,1729774	0,34732938	0,7016986	5,031513	0,285119065	No

NS_72E_1	Northern	*Skeletonema marinoi*	Yes	72	1	0,1	34,8359922	0,25666228	0,5048999	3,198158	0,335806592	No
NS_72E_2	Northern	*Skeletonema marinoi*	Yes	72	2	0,3	33,7802366	0,07160102	0,8182735	6,580721	0,080431039	No
NS_72E_3	Northern	*Skeletonema marinoi*	Yes	72	3	0,3	33,1814054	0,20366803	0,996021	9,908798	0,627557185	No
AS_72E_2	Southern	*Skeletonema marinoi*	Yes	72	2	0,1	30,4653842	0,11888413	0,8086233	6,436108	1,641207474	No
AS_72E_3	Southern	*Skeletonema marinoi*	Yes	72	3	0,1	31,4892349	0,26104781	0,5030783	3,184772	0,334401024	No
NS_120_1	Northern	*Skeletonema marinoi*	No	120	1	0,1	31,1064097	0,38090488	0,6173238	4,143085	0,41430845	No
NS_120_2	Northern	*Skeletonema marinoi*	No	120	2	0,2	27,5932223	0,36033446	1,6657548	46,31853	2,663315563	No
NS_120_3	Northern	*Skeletonema marinoi*	No	120	3	0,1	29,5903638	0,11773112	1,0697533	11,7423	6,282131692	No
AS_120_1	Southern	*Skeletonema marinoi*	No	120	1	0,1	27,6784401	0,41557011	1,6403235	43,68411	10,4841858	No
AS_120_2	Southern	*Skeletonema marinoi*	No	120	2	0,1	29,416249	0,35781407	1,1217139	13,23469	1,191122395	No

AS_120_3	Southern	*Skeletonema marinoi*	No	120	3	0,1	28,7454249	0,21251628	1,3219061	20,98486	6,400382401	No
NS_96E_1	Northern	*Skeletonema marinoi*	Yes	96	1	0,1	28,7774041	0,20923799	1,3123626	20,52875	6,774489034	No
NS_96E_2	Northern	*Skeletonema marinoi*	Yes	96	2	0,1	30,6686192	0,09929191	0,7479724	5,597221	3,358332473	No
NS_96E_3	Northern	*Skeletonema marinoi*	Yes	96	3	0,1	31,1076749	4,2397995	0,6169462	4,139484	0,703712296	No
AS_96E_2	Southern	*Skeletonema marinoi*	Yes	96	2	0,1	31,9006449	0,48697349	0,3803023	2,400503	0,820972163	No
AS_96E_3	Southern	*Skeletonema marinoi*	Yes	96	3	0,1	31,4667384	0,96359122	0,5097919	3,234386	0,452814075	No
NS_144_1	Northern	*Skeletonema marinoi*	No	144	1	0,3	31,6034476	0,21924324	0,4689941	2,944382	0,088331453	Yes
NS_144_2	Northern	*Skeletonema marinoi*	No	144	2	0,1	28,9694188	0,26131263	1,2550602	17,9912	1,25938414	No
NS_144_3	Northern	*Skeletonema marinoi*	No	144	3	0,1	27,021499	0,18399474	1,8363726	68,60766	15,09368554	No
NS_144_4	Northern	*Skeletonema marinoi*	No	144	4	0,1	27,671924	0,2818673	1,6422681	41,50619	0,968477841	No

AS_144_1	Southern	*Skeletonema marinoi*	No	144	1	0,1	27,7528655	0,2069242	1,6181129	6,833064	1,639935318	Yes
AS_144_2	Southern	*Skeletonema marinoi*	No	144	2	0,1	30,378287	5,54748106	0,8346155	5,02799	0,729058546	No
AS_144_3	Southern	*Skeletonema marinoi*	No	144	3	0,1	30,8246975	0,39641955	0,7013944	43,88015	8,337227679	No
NS_120E_1	Northern	*Skeletonema marinoi*	Yes	120	1	0,1	29,064181	0,41250336	1,2267806	16,85701	1,432845913	No
NS_120E_2	Northern	*Skeletonema marinoi*	Yes	120	2	0,1	30,5483348	0,30854112	0,7838686	6,07951	1,945443237	No
NS_120E_3	Northern	*Skeletonema marinoi*	Yes	120	3	0,1	29,9609804	2,06072736	0,9591511	9,1023	2,912736025	No
AS_120E_1	Southern	*Skeletonema marinoi*	Yes	120	1	0,1	32,066054	4,36664438	0,3309397	2,142593	0,728481717	No
AS_120E_2	Southern	*Skeletonema marinoi*	Yes	120	2	0,1	30,5243721	0,25907794	0,7910197	6,180444	1,977742172	No
AS_120E_3	Southern	*Skeletonema marinoi*	Yes	120	3	0,1	33,4756285	4,67993402	-0,089716	0,813363	0,065069014	No
