# Supplementary material for: Molecular diet analysis enables detection of diatom and cyanobacteria DNA in the gut of Macoma balthica
Source: PLoS One. 2022 Nov 23;17(11):e0278070. doi: 10.1371/journal.pone.0278070 (PMC9683582; doi:10.1371/journal.pone.0278070)
Supplement: S2 Table — Sample name is the Macoma balthica individual, biosample accession refers to the reference number to the sequencing data in the National Center for Biotechnology Information (NCBI) sequence read archive (SRA), region refers to phytoplankton fed refers to the origin of the individual, phytoplankton culture fed to the individual, empty guts refers to the treatment where the individuals were placed in fresh water for 24 hours to empty their guts, time hours refers to the number of hours after feeding that the individuals were sampled, and biological replicate refers to the individual replicate number. (PDF) [file pone.0278070.s006.pdf]

Sample name	Biosample accession	Region	Phytoplankton fed	Empty guts	Time hours	Biological replicate
NS0_1	SAMN27069735	Northern	*Skeletonema marinoi*	No	0	1
NN0_1	SAMN27069736	Northern	*Nodularia spumigena*	No	0	1
AS0_1	SAMN27069737	Southern	*Skeletonema marinoi*	No	0	1
N-24_1	SAMN27069738	Northern	None	No	-24	1
N-24_2	SAMN27069739	Northern	None	No	-24	2
N-24_3	SAMN27069740	Northern	None	No	-24	3
A-24_1	SAMN27069741	Southern	None	No	-24	1
A-24_2	SAMN27069742	Southern	None	No	-24	2
A-24_3	SAMN27069743	Southern	None	No	-24	3
AN0_1	SAMN27069744	Southern	*Nodularia spumigena*	No	0	1
NS48_1	SAMN27069745	Northern	*Skeletonema marinoi*	No	48	1
NS48_2	SAMN27069746	Northern	*Skeletonema marinoi*	No	48	2
NN48_1	SAMN27069747	Northern	*Nodularia spumigena*	No	48	1
NS24_1	SAMN27069748	Northern	*Skeletonema marinoi*	No	24	1
NN24_1	SAMN27069749	Northern	*Nodularia spumigena*	No	24	1
AS24_1	SAMN27069750	Southern	*Skeletonema marinoi*	No	24	1
AN24_1	SAMN27069751	Southern	*Nodularia spumigena*	No	24	1
NS0E_1	SAMN27069752	Northern	*Skeletonema marinoi*	Yes	0	1
NN0E_1	SAMN27069753	Northern	*Nodularia spumigena*	Yes	0	1
AS0E_1	SAMN27069754	Southern	*Skeletonema marinoi*	Yes	0	1
AN0E_1	SAMN27069755	Southern	*Nodularia spumigena*	Yes	0	1
AN48_1	SAMN27069756	Southern	*Nodularia spumigena*	No	48	1
NS72_1	SAMN27069757	Northern	*Skeletonema marinoi*	No	72	1
NN72_1	SAMN27069758	Northern	*Nodularia spumigena*	No	72	1
AS72_1	SAMN27069759	Southern	*Skeletonema marinoi*	No	72	1
AN72_1	SAMN27069760	Southern	*Nodularia spumigena*	No	72	1
NS144_1	SAMN27069761	Northern	*Skeletonema marinoi*	No	144	1
NN144_1	SAMN27069762	Northern	*Nodularia spumigena*	No	144	1
AS144_1	SAMN27069763	Southern	*Skeletonema marinoi*	No	144	1
AN144_1	SAMN27069764	Southern	*Nodularia spumigena*	No	144	1

1
