# Supplementary material for: Molecular diet analysis enables detection of diatom and cyanobacteria DNA in the gut of Macoma balthica
Source: PLoS One. 2022 Nov 23;17(11):e0278070. doi: 10.1371/journal.pone.0278070 (PMC9683582; doi:10.1371/journal.pone.0278070)
Supplement: S1 File — Within this experiment, we had an additional treatment to test the effectiveness of “gut clearing,” the practice of allowing macrofauna purge their guts in fresh water for 24 hours. This is a common practice in stable isotope studies, and is intended to remove any recently digested items from the analysis to not disrupt the signal, as stable isotope analysis is primarily interested in the assimilated food items [9–12]. The statistical methods used are described in the main text, and presented and discussed here. (PDF) [file pone.0278070.s007.pdf]

Within this experiment, we had an additional treatment to test the effectiveness of “gut clearing,” the practice of allowing macrofauna to purge their guts in fresh water for 24 hours. This is a common practice in stable isotope studies, and is intended to remove any recently digested items from the analysis to not disrupt the signal, as stable isotope analysis is primarily interested in the assimilated food items [1–4]. The statistical methods used are described in the main text, and presented and discussed here.

Gut clearing results

There was no difference in detected DNA between *M. balthica* with cleared guts or without in the *S. marinoi* treatments (S3A and S3C Fig; ANOVA, $F_{1,51}=0.49$, $p=0.49$). Additionally, no linear increase or decrease in abundance of DNA fragments was detected for either region with cleared or not cleared guts after consuming *S. marinoi*, with the exception of the increase in southern not cleared over time, as stated earlier (northern cleared: $R^2=-0.0403$, $F_{15,15}=0.38$, $p=0.55$; northern not cleared: $R^2=-0.027$, $F_{23,23}=0.36$, $p=0.55$; southern cleared: $R^2=-0.0068$, $F_{13,13}=0.0068$, $p=0.94$; southern not cleared: $R^2=0.21$, $F_{19,19}=6.3$, $p=0.021$). Gut clearing by itself was also not a significant explanatory factor for *N. spumigena* (S3B and S3D Fig; ANOVA, $F_{1,50}=0.064$, $p=0.8$), but interacted with region significantly (ANOVA, $F_{1,50}=6.7$, $p=0.013$). The southern clams unexpectedly showed an increase in *N. spumigena* DNA when placed in fresh water for 24 hours, but the northern clams decreased. All intercepts were significant in the linear models for *N. spumigena* for both regions, with and without gut clearing, but there was no significant trend over time (northern cleared: $R^2=-0.061$, $F_{16,16}=0.022$, $p=0.88$; northern not cleared: $R^2=0.079$, $F_{20,20}=2.81$, $p=0.109$; southern cleared: $R^2=-0.011$, $F_{13,13}=0.84$; $p=0.38$; southern not cleared: $R^2=-0.043$, $F_{21,21}=0.092$; $p=0.76$).

Gut clearing effectiveness discussion

Gut clearing of macrofauna is a common method when evaluating prey signals through stable isotope measurements to remove recently eaten material, as stable isotope analysis measures the tissue-incorporated prey [1–4]. We detected phytoplankton DNA by qPCR in all samples subjected to gut clearing, indicating that the guts are still at least partially full, and potentially influencing the stable

isotope analysis. The regions did not differ in detection of the diatom *S. marinoi*, but did differ in trends of detection of *N. spumigena* with gut clearing. The northern clams feeding on *N. spumigena* was the only one that followed our predictions, decreasing in DNA detected when subjected to gut clearing. However, the southern clams feeding on *N. spumigena* defied our predictions and general understanding of trophic ecology and increased in phytoplankton DNA detected over time with gut clearing, despite no new phytoplankton sources. Our only explanation, other than a fault in the method, is that the *M. balthica* are consuming their own feces and we are still detecting the DNA signal from twice-consumed prey phytoplankton. Indeed, both *M. balthica* and other detritivorous species consuming their own feces has been documented previously, primarily as a method to consume bacteria which are “farmed” [5,6]. Detritivores are known to derive part of their diet from feces [7], which can be especially beneficial when in a period of low fresh organic matter input from the pelagic zone. From modelling, it has been shown that *M. balthica* could deplete its yearly sediment food bank by autumn [8], creating a food-limitation that consuming feces could help rectify in the short-term. This creates difficulties when attempting to understand the diet of detritivores, as current methods are limited, with stable isotopes not able to capture the consumption of material that has a similar signal to the organism, and molecular methods unable to give a timeframe of when the material was consumed.

References

1. Guiguer KRRA, Barton DR. The Trophic Role of Diporeia (Amphipoda) in Colpoys Bay (Georgian Bay) Benthic Food Web: A Stable Isotope Approach. Journal of Great Lakes Research. 2002;28: 228–239. doi:10.1016/S0380-1330(02)70579-0

2. Feuchtmayr H, Grey J. Effect of preparation and preservation procedures on carbon and nitrogen stable isotope determinations from zooplankton. Rapid Commun Mass Spectrom. 2003;17: 2605–2610. doi:10.1002/rcm.1227

3. Smyntek PM, Teece MA, Schulz KL, Thackeray SJ. A standard protocol for stable isotope analysis of zooplankton in aquatic food web research using mass balance correction models. Limnol Oceanogr. 2007;52: 2135–2146. doi:10.4319/lo.2007.52.5.2135

4. Bergamino L, Lercari D, Defeo O. Food web structure of sandy beaches: Temporal and spatial variation using stable isotope analysis. Estuarine, Coastal and Shelf Science. 2011;91: 536–543. doi:10.1016/j.ecss.2010.12.007

5. Newell R. The role of detritus in the nutrition of two marine deposit feeders, the prosobranch *Hydrobia ulvae* and the bivalve *Macoma balthica*. Proceedings of the Zoological Society of London. 1965;144: 25–45. doi:10.1111/j.1469-7998.1965.tb05164.x

6. Hylleberg J. Selective feeding by *Abarenicola pacifica* with notes on *Abarenicola vagabunda* and a concept of gardening in lugworms. Ophelia. 1975;14: 113–137. doi:10.1080/00785236.1975.10421972

7. Dethier MN, Hoins G, Kobelt J, Lowe AT, Galloway AWE, Schram JB, et al. Feces as food: The nutritional value of urchin feces and implications for benthic food webs. Journal of Experimental Marine Biology and Ecology. 2019;514–515: 95–102. doi:10.1016/j.jembe.2019.03.016

8. Ehrnsten E, Norkko A, Timmermann K, Gustafsson BG. Benthic-pelagic coupling in coastal seas – Modelling macrofaunal biomass and carbon processing in response to organic matter supply. Journal of Marine Systems. 2019;196: 36–47. doi:10.1016/j.jmarsys.2019.04.003
