# Supplementary material for: Molecular diet analysis enables detection of diatom and cyanobacteria DNA in the gut of Macoma balthica
Source: PLoS One. 2022 Nov 23;17(11):e0278070. doi: 10.1371/journal.pone.0278070 (PMC9683582; doi:10.1371/journal.pone.0278070)
Supplement: Attachment — Submitted filename: Response_to_reviewers.pdf [file pone.0278070.s009.pdf]

Thank you for submitting your manuscript to PLOS ONE. I have received feedback from the two reviewers on the revised version of the paper, and Reviewer #1 has made some additional suggestions that I think should improve the manuscript and I ask that you consider those suggestions where it is practical. Consequently, I invite you to submit a revised version of the manuscript that addresses the minor revisions that the reviewer recommends. I realize that there are questions that might be challenging to address with the data in hand, but I expect to be able to recommend acceptance of the paper once these points the reviewer makes have been acknowledged and the constraints on using the approach you and your colleagues have undertaken are clearly spelled out. I thank you again for considering PLOS One to publish your results and look forward to receiving a second revised version of the paper. I am currently on a research cruise and have some limits to my email access for the next couple weeks, so please be patient with my response time if you are able to return the manuscript in that time frame.

Dear Dr. Cooper,

Thank you for the opportunity to submit our revised manuscript. We are very glad to more clearly communicate the constraints as well as the strengths of our data, and are appreciative of reviewer 1's comments. The reviewer has helped point out the less unsupported claims that we have made with our data, as well as highlighting some interesting and unexpected results of our study that will appeal to a broader audience. We have stepwise addressed all the reviewer's comments and made changes to the manuscript, as well as supplementary information. Please find replies to the reviewer comments, as well as manuscript text changes and referenced line numbers in the tracked changes version of the manuscript.

We are very excited about the prospect of publishing our manuscript with PLOS ONE.

Sincerely,

Julie Garrison and co-authors

Reviewer #1: This manuscript is much improved, and the inferences presented more reasonable. The paper is still written to be mainly about methodology. However, given that the experiments cannot indicate the duration of detectability of DNA in bivalve guts after consumption, the main methodological conclusion is simply that DNA can be detected in the guts, which was not really questionable beforehand. Nevertheless, your results have important implications for ecology and the body of work on deposit-feeder food sources that could be emphasized more clearly.

Thank you for reviewing our manuscript again. We appreciate the time that you have taken, and agree that our manuscript focuses on methodology and an aim that we could not meet. We have revised the manuscript to focus more broadly on deposit-feeder diet, specifically altering the end of the abstract to focus on the DNA metabarcoding diet results (lines 39-43), the first paragraph of the discussion to focus on your conclusion 3 from the next comment (lines 366-371), and recommending future studies to fill the gaps left by our study (lines 433-435 and 438-440). We hope that you find these changes adequate to redirect our manuscript from a methodological study to an exploratory one.

Lines 39-43: "However, DNA metabarcoding of the 23S rRNA phytoplankton gene found in the clam guts showed that added food (i.e. N. spumigena and S. marinoi) did not make up a majority of the detected diet. Our results suggest that these detritivorous clams therefore do not react as quickly as previously thought to fresh organic matter inputs, with other phytoplankton than large diatoms and cyanobacteria constituting the majority of their diet."

Lines 366-371: "This could be a result of too little time to react to the fresh organic matter, which was 24 hours in our study, and perhaps varied access time to fresh food should be evaluated in future experiments. Additionally, we found that phytoplankton DNA in sediments can remain viable and ingested by benthic detritivores for at least six months following organic matter input, which can have important implications for the understanding of benthic detritivore feeding ecology."

Lines 433-435: "This finding furthers our hypothesis that the M. balthica from different stations have different trophic strategies, but could also simply be an indication of different phytoplankton in the sediment, and would require analysis of the phytoplankton DNA present in the sediment before the additions of organic matter made in our experiment to make accurate conclusions."

Lines 438-440: "Interestingly, our study indicates that phytoplankton DNA remains available in the sediment in ingestible form for at least six months after fresh organic matter addition, and is consumed by benthic detritivores."

In this study, DNA in the clams' guts before the experiment indicated that, even after 9 months without adding phytoplankton, Macoma were still consuming phytoplankton or at least bacteria that had consumed phytoplankton-derived material. When phytoplankton of particular taxa were then added in substantial amounts to the sediments, there was no increase in relative occurrence of the DNA of those taxa in the clams' guts from before additions to the end of the experiment (1 week), and these added taxa remained a minor component of gut contents. If that assessment of your results is inaccurate, please state the results more simply and clearly for the many readers on this topic who have not used these methods themselves.
Although not what the experiment set out to investigate, from an ecological perspective the major conclusions appear to be that
(1) The DNA of phytoplankton can remain available in the sediments in ingestable form for at least 6 months after cessation of any additions. Whether this DNA is contained within persistent phytoplankton cells or bacteria that consumed phytoplankton-derived material is unclear.
(2) The levels of phytoplankton-derived material persisting after 6 months can sustain deposit-feeding Macoma.
(3) Pulsed additions of fresh phytoplankton do not alter the clams' diet within 7 days. This finding

might indicate that the clams do not respond within a week to the availability of added material, or that added material requires a period of processing via bacteria or passing through the guts of deposit-feeders before becoming viable foods.
Conclusion #3 is curious, as previous studies have shown that even if deposit-feeders do not assimilate the fresh material, they do in fact ingest it, so the DNA from the fresh material should have been detectable on the clams' guts.

We have modified the manuscript to better reflect the three conclusions underlined by the reviewer. We agree without exception with your first two conclusions presented here from our work, and we have highlighted these conclusions in the discussion of our manuscript (lines 366-371, 433-435, and 438-440, all mentioned above). We also agree mostly with your third conclusion, with the provision that the clams only had access to the fresh organic matter for 24 hours, giving support to your hypothesis that Macoma balthica requires a period of processing before it can process freshly deposited phytodetritus as food. We have added this information to the discussion section in lines 366-371. As discussed in multiple places within the manuscript, this is the first study that we are aware of that investigates benthic detritivore diet by molecular methods, and multiple improvements can be made in future work. We are happy to make recommendations for future work on this topic, and hopeful that future researchers will be able to learn from our mistakes. We also agree that our results are interesting and open up a number of questions, which hopefully will be evaluated in future work. While our data did not support our original aims, we have found support for these conclusions that are perhaps more broadly interesting than the study that we set out to perform.

All three conclusions have been highlighted in our conclusions section, which now reads "Our results suggest that the feeding of two regional groups of the Baltic clam on a diatom species do not differ, but clams from the more sheltered site fed significantly more on a cyanobacteria species, possibly indicating higher availability of cyanobacterial material or higher trophic plasticity that results in potential variations of feeding strategies. However, these fresh organic matter additions did not make up the majority of the clams' diet. Our experimental data suggests a significant proportion of the diet is made up of picocyanobacteria and unicellular green algae, which were not added freshly during the experiment. This indicates that phytoplankton DNA can remain in sediments for at least six months, and that these phytoplankton can sustain deposit-feeding Macoma balthica" in lines 470-479.

1. L 35-36. If I understand your results correctly, this statement is not justified. The relative occurrence of neither N. marinois or N. spumigena increased in the clams' guts from before to after feeding, so there is no confirmation that DNA from the added material per se was still present in the guts at the end of the week-long experiment. DNA from these taxa was present in the sediments and already being consumed in similar amounts before the addition, so we don't known that the clams were ingesting the added material as opposed to what was already there. If your data do show that they were clearly using newly added material, please point out those data and make the point more plainly in the Results to refute the alternative possibility.

This sentence has been modified to read "Interestingly, the cyanobacteria and diatom DNA fragments were still detectable by qPCR in the guts of M. balthica one week after removal from its food source. However, DNA metabarcoding of the 23S rRNA phytoplankton gene found in the clam guts showed that added food (i.e. N. spumigena and S. marinoi) did not make up a majority of the detected diet. Our results suggest that these detritivorous clams therefore do not react as quickly as previously thought to fresh organic matter inputs, with other phytoplankton than large diatoms and cyanobacteria constituting the majority of their diet" in lines 37-43. We agree that we cannot be certain that the clams are indeed consuming the added fresh phytoplankton and not aged material from the original sediments (but see our response to comment 10), so we have modified to point out more of our findings from the DNA metabarcoding results and highlight your conclusion 3 from the previous comment.

2. L 58. Is reference [14] a thesis? If so, say so in the reference list. If not, without more information it is not apparent how to obtain this document. Only references that are generally available should be cited. Is there perhaps a doi number?
Yes, reference [14] is a PhD thesis, and has been updated in the references to make it clear. This thesis is available, and a link has been added to aid readers to find it.

14. Hedberg P. Responses of benthic-pelagic coupling to environmental change. PhD thesis, Stockholm University. 2021. Available: http://su.diva-portal.org/smash/record.jsf?pid=diva2%3A1594570&dswid=-3644

3. L 116. "physical forcing influences on their feeding strategy". The meaning of this phrase is unclear. Just say "differing availability of the two phytoplankton species". You don't know whether bloom dynamics, current patterns, or water-column processes (buoyancy, predation by zooplankton) determine their relative availability, so just stick with the proximate factor that the animals will actually respond to.

We agree with the reviewer's assessment that we cannot be sure that differences in feeding between the two groups of clams would be due to physical forcing. We have revised this sentence to read "ii) if feeding by the two M. balthica regional groups on each of the phytoplankton species differs, which would likely be due to trophic plasticity and differing availability of phytoplankton on their feeding strategy" in lines 122-125.

4. L 118-119. This phrasing begs the question of why the southern population would exhibit higher deposit-feeding, as this prediction has no basis in preceding text. Deposit-feeding is not an inherently more profitable feeding mode than filter-feeding. The difference must result from variations in food availability accessible by the different feeding modes. I suggest the wording "... southern population, with longer open-water period and settlement of greater phytoplankton biomass (reference), would have higher feeding rates."

We agree that this statement was not adequately supported. We have revised the text to better connect reference [66] with this statement, where clams in sheltered habitats were found to spend more time deposit feeding than clams from exposed habitats. Since the phytoplankton was mixed into the sediment in the experiment, the sheltered southern clams should have higher feeding magnitudes due to their preference for deposit feeding. The sentence now reads "We predicted that both phytoplankton species would be consumed by all clams, but that S. marinoi would be consumed at a higher magnitude due to the higher nutritional value, and that the sheltered southern clams would have higher phytoplankton consumption due to higher deposit feeding, where the phytoplankton in this experiment could be found" in lines 125-129.

5. L 172-173. Please state explicitly that the -24 clams were not exposed to the freshly amended sediments before being sampled, if that was the case. Bivalves can respond almost immediately to food additions (within 20 min), although they do not always do so depending on prior conditions.

Also, if you have the data, please state the range of shell lengths of clams added to the mesocosms.

Yes, your assessment is correct that -24 h clams were not exposed to fresh phytoplankton, and we have tried to make this more clear in lines 184-185, but would also like to point out that this information was already available in lines 198 and figure 1. We agree that the literature indicates that bivalves can respond almost immediately to fresh food additions, and have included the suggestion to take samples sooner after feeding than 24 hours in our conclusions (lines 366-371; mentioned in previous replies). We did not measure precisely the shell lengths of individual clams, but

have added the range 10 to 20 mm to our methods (lines 200-202) and emphasize the wet weight available in Table S1. The text now reads "For M. balthica wet weight, please see S1 Table. While individual shell lengths were not measured, all clams were between 10-20 mm to reduce variation by life stage and particle size processing limitations."

6. L 273-274. Although there was no decrease, there was also no increase at the beginning or throughout the experiment, which is perhaps your most interesting finding. The clams were already consuming DNA of the experimental phytoplankton taxa at comparable rates before the experimental additions.

Yes, you are correct. We have altered this sentence to highlight the lack of increase as well, and it now reads "Additionally, there were no significant differences in phytoplankton DNA concentrations detected between sampling time points for either S. marinoi or N. spumigena (Table 1), indicating no significant increase or decrease in phytoplankton DNA detected over time" in lines 286-288. We have also highlighted this finding in our discussion section, as mentioned in a previous response.

7. L 275. Another striking aspect is that the variation among replicates in consumption of the added diatom taxon increased greatly toward the end of the experiment in both regions. Although response times certainly vary, some experiments have revealed that deposit-feeders can take at least a week to react to additions of phytoplankton (e.g. Deep-Sea Res I 55:1503, 2008). It is possible that the increased variation in consumption of S. marinoi toward the end of your experiments indicated that some individuals were just starting to respond to the food addition from a week before.

Yes, we also have noticed the large variation between replicates, but since the difference between timepoints in ingestion of diatoms was not statistically significant we cannot be certain that it is actual biological variation and not methodological, or a result of only three replicates per time point and treatment. We have suggestions to future research both to extend the time that molecular monitoring of diet occurs in line 374, and to increase numbers of replication in line 383. The higher replication would allow future experiments to tease out if the large variation is due to individual diet differences or limitations of the methods, and longer experiment times will hopefully allow for a decline in detected amended phytoplankton.

8. L 283. Please increase the font sizes of the axis labels in Fig. 4, as they are currently too small to be read easily.

The font sizes in figure 4 have been increased.

9. L 301-304. The higher level of N. spumigena DNA in the guts of the southern population was evident before any experimental addition (after 6 mo without additions of food), and the rate of feeding on N. spumagena was unaffected by additions. It appears that the N spumagena DNA was already available at a higher level in the sediments from the southern population before the experiments began.

Yes, the southern clams started with higher N. spumigena than the northern clams, even without additions of fresh material. The southern clams were even without new food sources for 3 months longer than the northern clams, indicating that the clams were possibly actively avoiding N. spumigena in the sediment. However, we do see an increase from the baseline -24 value after 48 and 72 hours in the southern clams. In contrast, the northern clams demonstrated an immediate increase already at 0 h from the baseline, and a sharp increase after 48 and 144 h.

In accordance with the results, we have added to the results and discussion sections cautioning against over interpretation of our results. The results reads "However, the southern clams contained significantly higher ($F_{1,50}$ = 23.5, $p < 0.001$) amounts of N. spumigena 16S rRNA gene fragments in

their guts than the northern clams (Fig 2B; Table 1), but the southern clams showed already higher baseline values at -24 h (Fig 2B)" in lines 317-320. The discussion section now reads "Additionally, sediment samples from before, during, and after the experiment would be required to confirm the targeted phytoplankton DNA levels in the sediment. Indeed, the baseline of N. spumigena in the southern clams was higher than the northern clams before fresh phytoplankton were added, and these results must be taken with that consideration" in lines 414-418.

10. L 353-354. If you did not see an increase after vs. before phytoplankton additions, why would you expect to see a decrease? The clams were already subsisting on phytoplankton (or bacteria that had consumed phytoplankton material) before the experiment started, and continued to feed on the same phytoplankton-derived material that was already present in the sediments after feeding. There was no detectable change in diet after phytoplankton additions.

This section in lines 372-384 of the discussion serves to explain some potential reasons why we did not see an increase after feeding or a linear decrease as seen in previous studies, i.e. too few sampling points, sampling points not close enough to the feeding, or far enough from the feeding, or too few replicates or any other potential reasons. Out of our two groups of M. balthica and two phytoplankton species, we did see two out of four treatments increase (although statistically insignificant) from the baseline following feeding: northern clams feeding on S. marinoi and northern clams feeding on N. spumigena. When also including the emptied gut treatments presented in the supplementary material, we find one additional treatment where feeding increased phytoplankton DNA detection from the baseline: southern clams feeding on N. spumigena with emptied guts. We cannot be sure why all of our treatments didn't increase from the baseline following feeding, and this should be investigated in future studies. Additionally, of these 3 treatments which showed an increase following feeding, none of them showed a linear decrease from the initial increase.

We have added the lack of increase following feeding to our first sentence of this paragraph, which now reads "Unlike previous studies that looked at the prevalence of prey DNA in e.g. insect and spider consumers [25,29,33,35–38], we found no consistent increase in phytoplankton detection or linear decrease in the guts of the clam M. balthica following feeding" in lines 372-374.

11. L 419-420. Although Macoma can affect meiofauna via trophic competition, I am unaware of studies showing actual consumption of meiofauna by Macoma. I would use another organism as an example here (juvenile fish commonly eat meiofauna), and then stick with Macoma consuming bacteria in subsequent text.

We have added a reference to this sentence to Ólafsson et al. 1993 [52], which shows that M. balthica consumes harpacticoid copepods in line 448. This finding comes from a feeding experiment, where a reduction in number of harpacticoid copepods was observed when sharing a habitat with M. balthica, and thus has limitations, but is widely cited as evidence of M. balthica feeding on meiofauna. We also believe that a study with newer molecular methods would be beneficial.

12. L 434-435. Replace "by accident" with "incidentally"

The suggested change has been made in line 466.

13. L 442. The phrase "high trophic plasticity from potentially variations of feeding strategies" is awkward and has unclear meaning. The most logical explanation is that cyanobacterial material was simply more available in the sediments from the southern region. With planktonic larvae, Macoma from sampling regions so close together are likely all from the same population genetically, and they are not necessarily varying their feeding mode. For Macoma in the southern sediments that consumed greater amounts of cyanobacterial material before the addition experiments, did you notice that they

had their siphons extended more often? If not, it appears that feeding mode did not explain the difference in consumption of cyanobacteria.

The phrase has been revised to "Our results suggest that the feeding of two regional groups of the Baltic clam on a diatom species do not differ, but clams from the more sheltered site fed significantly more on a cyanobacteria species, possibly indicating higher availability of cyanobacterial material or higher trophic plasticity that results in potential variations of feeding strategies" in line 470. Unfortunately, no reliable observations of behavior were made during the experiment. We include the word "potential" to highlight that we did not observe this difference in feeding strategy during our experiment, but found it a likely explanation for our results. Of course, additional studies should be conducted to investigate this, and we recommend to observe the feeding strategies, by for example observing time spent with siphons extended and providing food in different methods (mixing into sediment vs. sedimenting from the water column vs. sediment surface).

More generally, I would avoid language implying that Macoma from the two sampling regions were in fact different "populations". The sampling regions are quite close together, and Macoma has pelagic larvae that could easily disperse between these areas. I doubt that there are genetic differences between them, and a number of readers think of "populations" as being geographically or genetically differentiated. I would avoid reference to the "two populations" and rely on the term "regions".

We have revised the manuscript to avoid the term "population" when describing clams from the two regions. Instead, we have followed your suggestion and used "northern clams," "southern clams," and "regions" to describe these groups. Changes have been implemented throughout the manuscript and in figures 3 and 4, as well as supplementary information.

14. L 445. From the perspective of "ecological" (food web) models, the term "feeding rate" here just means diet, as your "feeding rate" data simply indicate relative proportions of different foods consumed. The DNA data do not indicate absolute amounts (in mass) of different foods consumed per unit time, which is the way "feeding rate" in trophic analyses is defined. Thus, in relating your data to ecological models, the terms "diet" and "feeding rate" are redundant. In this paper you are trying to appeal to people concerned with trophic analyses, so I would come up with a different term than "feeding rate" to avoid confusion.

We agree that "feeding rate" is not the most accurate term that could be used here, and have made changes throughout the manuscript to instead refer to this detection of food over time as simply "feeding" or "feeding magnitude."
